# Role of Copper Oxide on Epoxy Coatings with New Intumescent Polymer-Based Fire Retardant

**DOI:** 10.3390/molecules25245978

**Published:** 2020-12-17

**Authors:** Riyazuddin, Samrin Bano, Fohad Mabood Husain, Jamal Akhter Siddique, Khadijah H. Alharbi, Rais Ahmad Khan, Ali Alsalme

**Affiliations:** 1 Department of Food Science and Nutrition, King Saud University, Riyadh 11451, Saudi Arabia; samriyaz5@gmail.com (R.); fhusain@ksu.edu.sa (F.M.H.); 2 Center of Chemical Sciences and Technology, Institute of Science and Technology, JNTU, Hyderabad 500085, India; samrinbano384@gmail.com; 3 Department of Chemistry, Aligarh Muslim University, Aligarh 202002, India; 4 Department of Chemistry, Science and Arts College, Rabigh Campus, King Abdulaziz University, Jeddah 21911, Saudi Arabia; khalharbe@kau.edu.sa; 5 Department of Chemistry, College of Science, King Saud University, Riyadh 11451, Saudi Arabia; krais@ksu.edu.sa

**Keywords:** epoxy coatings, charring-foaming agent, copper oxide, synergistic effect

## Abstract

Epoxy resins (EP) have been used as a thermos-setting material in the field of coating, casting, bonding agent, and laminating. However, a major drawback associated with its use is the lack of good flaming properties, and it is responsible for heavy smoke along with hazardous gases considerably limiting its uses in various fields. In this study, *N*-ethanolamine triazine-piperizine, a melamine polymer (ETPMP), was established as a new charring-foaming agent and was successfully synthesized with ethanolamine, piperizine, cyanuric chloride, and melamine as precursor molecules via the nucleophilic substitution reaction method. Elemental analysis and Fourier transform infrared (FTIR) spectroscopy analysis were applied to approve the synthesis of ETPMP and confirmation of its structure and characterization. The epoxy coating of intumescent flame retardant (IFR) was equipped by introducing ETPMP, ammonium polyphosphate (APP), and copper oxide (CuO) in multiple composition ratios. CuO was loaded at various amounts into the IFR-coating system as a synergistic agent. The synergistic action of CuO on IFR coatings was scientifically examined by using different analytical tests such as vertical burning test (UL-94V), limited oxygen index (LOI), thermal gravimetric analysis (TGA), cone calorimeter, and scanning electron microscope (SEM). The results showed that small changes in the amount of CuO expressively amplified the LOI results and enhanced the V-0 ratings in the UL-94V test. The TGA data clearly demonstrate that the inclusion of CuO can transform the thermal deprivation behavior of coatings with a growing char slag proportion with elevated temperatures. Information from cone calorimeter data affirmed that CuO can decrease the burning factors by total heat release (THR) together with peak heat release rate (PHRR). The SEM images indicated that CuO can enrich the power and compression of the intumescent char that restricts the movement of heat and oxygen. Our results demonstrate a positive influence of CuO on the epoxy-headed intumescent flame retardant coatings.

## 1. Introduction

Epoxy resin (EP) has been used as a thermos-setting material in the fields of coating, casting, bonding agent, and laminating [1,2,3]. However, it lacks good flaming properties and creates heavy smoke with noxious gases when enflamed, considerably limiting its uses in various fields [4]. It has been reported that most fire deaths are due to oxygen deficiency and poisonous gases instead of burns [5,6]. A demographical study of nearly 5000 deaths showed that the most common fire casualties are because of carbon monoxide (CO) injury [7]. Consequently, decreasing fume toxicity has become a vital concern in the development of flame retardancy of epoxy resin. Presently, wood and steel have been used at a large scale as a raw material for the construction of buildings, bridges, railways, and interior infrastructures. However, wood and steel are inherently flammable which effectively creates fire, disasters and mortalities. To minimize such issues and to retain fire safety, it is necessary to develop a fire-retardant coating for the protection of wood. 

Fire-resistant halogenated coatings are widely used, but these are associated with air pollution as they release hazardous gases. Therefore, an alternative non-halogen fire-resistant coating is the need of the hour. Two of the most commonly available non-halogen coatings are: (1) borate coatings and (2) magnesium/aluminum-controlled coatings. These coatings are effective fire-protectors as they form an opaque shielding char that minimizes the volume of fumes. On the contrary, these coatings mostly do not find practical applications because they are composed of heavy solids and their behavior in relation to binding agents is unclear [8]. Thus, more suitable, safe, stable non-halogen fire-resistant materials are needed.

The intumescent flame retardant (IFR) technology having quality features as not releasing toxic gases on any burning conditions are widespread in current research scenario. Such coatings are very much popular and impactful while applied on the steel and wood materials. In general, the Fire retarded coating layers having three basic constituents as per their functionality that are as follows; (a) the acid source, (b) the foaming agent, and (c) the charring agent [9,10]. The Shielding char releases nitrogen and ammonia on burning that are very hazardous volatile gases [11].During such type of conditions ammonium poly-phosphate (APP) behave as acid source, on other part pentaerythritol (PER) functions as a charring agent while melamine (MEL) act as a foaming agent. The contributions of these three constituents jointly considered as older version of IFR system. Number of researchers already claimed that the practice of such an unfashionable IFR system is not capable of handling fire efficiently and exhibits deprived anti-oxidation and fire protection behavior [12]. Furthermore, PER is a smaller substance that is straightforwardly affected by humidity and fallouts in underplaying action during fire extinguishing.

To overcome such problems, researchers have made numerous attempts to produce materials that have both charring and foaming characteristics. In this context, the triazine molecule was selected to play a role as a charring-foaming producer with the ability to acquire a great quantity of carbon and nitrogen components [13,14,15]. Moreover, some synthesized triazine derivatives show good thermal stability [16,17,18]. It has also been described that a few of the derived triazine produces polymers have shown less thermal constancy. Producing a thermally stable polymer which can protect the material from burning at high temperatures (more than 500 °C) is one of the major goals. In order to increase the power and compression of the intumescent char, a few synergistic molecules, such as zeolite [19,20,21], and metal derivative compounds [10,22,23] were thoroughly scrutinized. These have shown synergistic behavior, accelerating the intumescent process and establishing the compression of char. 

Copper oxide (CuO) is an inorganic crystal appearing as a red compound that is ecofriendly, having multiple catalytic applications and non-toxic on an industrial scale. Because of its acceptable catalytic properties, CuO is a remarkable substitute for metal catalysts [24,25]. Based on the catalytic uses of CuO, it is categorized as a synergist and is presumed to have a positive outcome on fire retardancy for IFR systems.

This work is in the continuation of previous research based on fire retardant system examined [19]. In current work we examine the relevance of CuO on epoxy headed intumescent flame retardant coatings. Different quantities of CuO were introduced into the epoxy/IFR coating method, and the impact of CuO on epoxy/IFR coatings were explored with the limited oxygen index (LOI), cone calorimeter test, thermogravimetric analysis (TGA), vertical burning test (UL-94V), and scanning electron microscopy (SEM). 

## 2. Results and Discussion

### 2.1. Fourier Transform Infrared Spectroscopy of ETPMP

**Figure 1:**
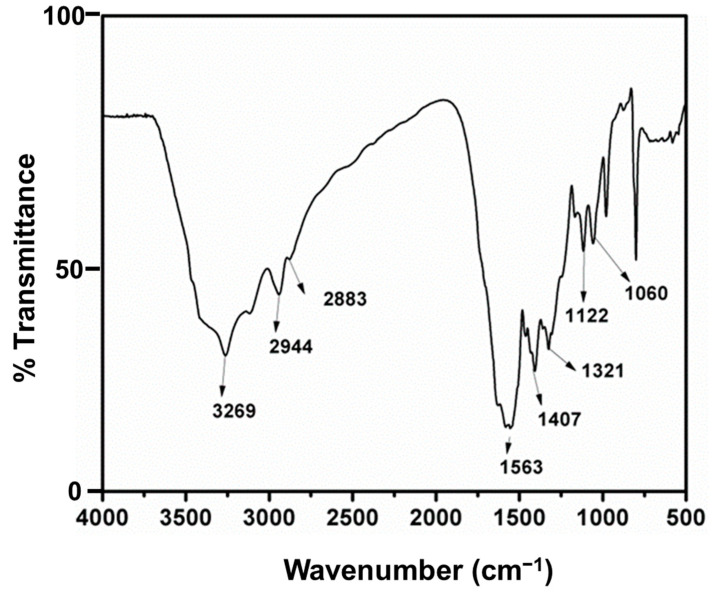
FTIR spectrum of ETPMP.

Figure 1 shows the FTIR spectrum of ETPMP. A broad adsorption peak is found at 3200–3450 cm^−1^ for N-H and O-H bonds-stretching patterns, respectively. The attributed peaks at 2944 cm^−1^ and 2883 cm^−1^ are for C-H bond-stretching interactions in the –CH_2_-CH_2_– group. The corresponding peaks at 1563 cm^−1^, 1407 cm^−1^, 1321 cm^−1^, and 1060 cm^−1^ are for ν_tr_, δ _O-H_ ν _tr-N_, and ν _C-O_, respectively. The peak at 850 cm^−1^ is absent in this spectrum, while one is specific for the C-Cl bond. Hence, we concluded that all three chlorine atoms of cyanuric chloride are successfully involved in this substitution. Our result is in accordance with a study demonstrating similar FTIR spectra for ETPMP [26].

### 2.2. Elemental Analysis

The ETPMP polymer elemental composition data is tabulated in Table 1. In this table, the calculated values and found values of C, N, O, and H are consistent. These results also confirm that ETPMP is formed. 

### 2.3. Thermal Degradation Behavior of ETPMP

**Figure 2:**
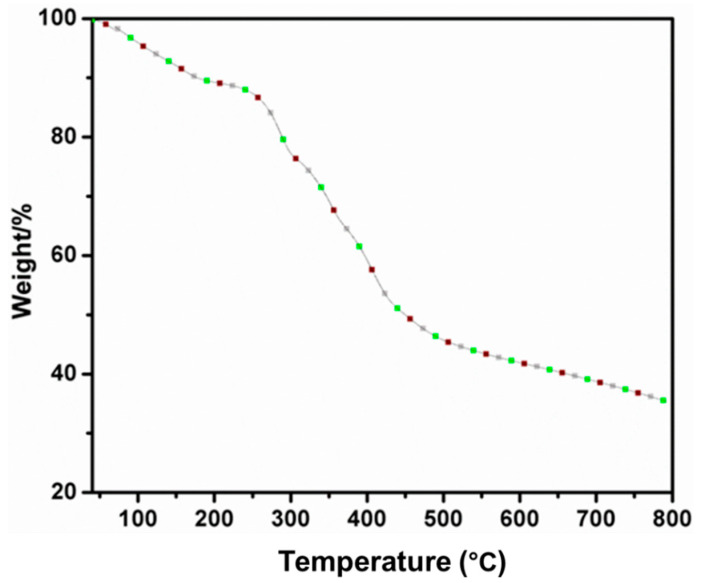
TGA curve of ETPMP.

TGA data provide information about the thermal stability of a polymer. Figure 2 and Table 2 represent the thermal breakdown of our synthesized polymer up to 800 °C. The TGA curve of ETPMP shows 35.58% of the char residue at 800 °C, indicating that during thermal degradation, the polymer produces a compact char that is thermally stable even at high temperatures. Here, we can see that the polymer mainly degraded in two stages. In the initial stage, which is at 90–200 °C, around 10% mass loss is observed because of the release of ammonia gas and water. In the later stage, a great mass loss (45%) of polymer was observed at 280–560 °C. At higher temperatures, cross-linkages in ETPMP were broken to release inert nitrogen and ammonia gases. During this process, a well-swollen char was formed, which inhibited the flow of heat and oxygen. 

### 2.4. Flame Retardancy Analysis

Table 3 represents the flame retardancy results of the prepared samples. A pure epoxy resin was shown to have high flammability, with 21% of LOI value, whereas with the introduction of IFR content to a pure epoxy resin, a great increment in LOI value of 28.7% was achieved. This result may be due to the fact that during the burning process, APP undergoes decomposition to produce phosphoric acid, which further initiates the dehydration of the ETPMP to produce the stable intumescent phosphor-carbonaceous intumescent char. From these results, it can be observed that the addition and increase of CuO up to 5% can increase the LOI values. This may be because the addition of CuO speeds up the phosphor-esterification and forms cross-linkages between the APP and IFR systems to make the char denser. The data also show that the addition of more than 5% of CuO to the coatings resulted in incompatibility and decreased LOI values. It might be due to the loss of compatibility between metal oxide (CuO) and coating system. Due to this incompatibility, CuO was unable to perform its catalytic activity. At higher loadings, metals and metal oxides shows incompatibility with coating material and remains separately.

The UL-94V test data of the samples show that, except for pure coating, all of the samples meet V-0 (high flame retardant) ratings. From LOI and UL-94V results, it became clear that incorporation of CuO can significantly enhance the flame retardant activity until its optimum loading concentration.

### 2.5. Thermogravimetric Analysis

**Figure 3:**
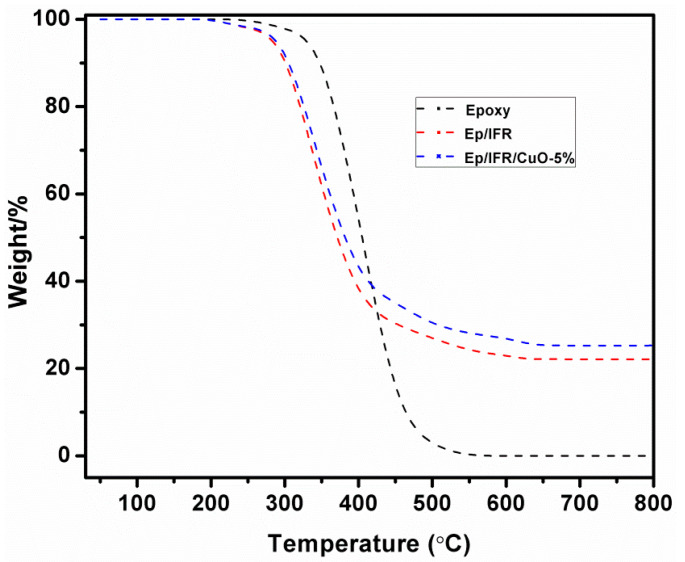
TGA curves of epoxy, Ep/IFR, and Ep/IFR/CuO-5% samples.

**Scheme 1:**
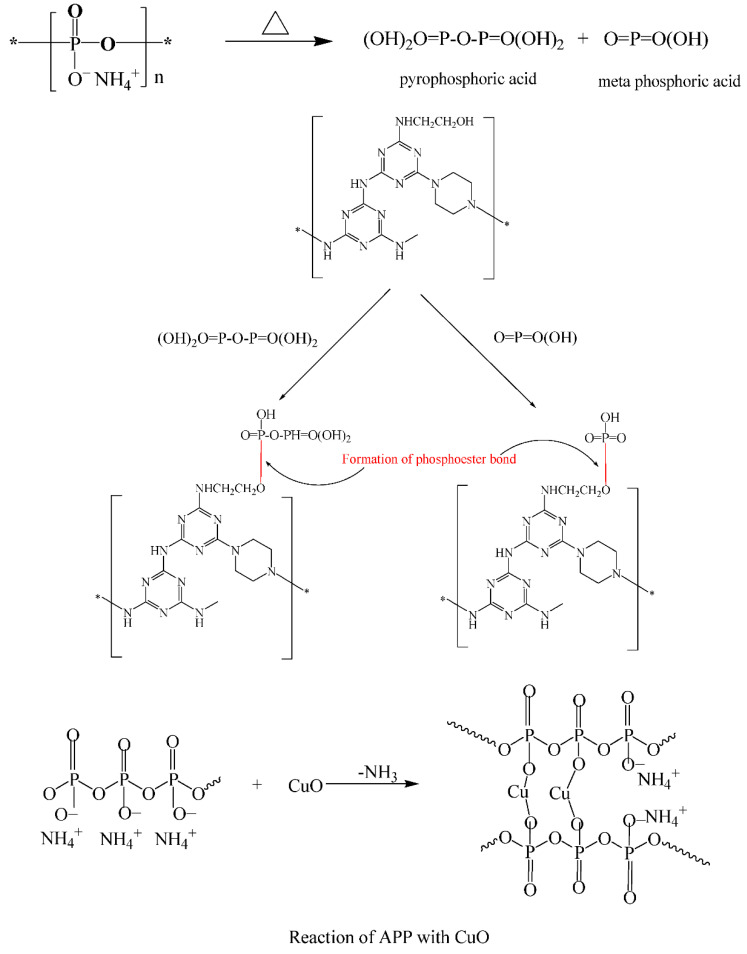
Thermal degradation pattern of the IFR system and CuO. Note: * represent the repeating unit of same molecule.

**Figure 4:**
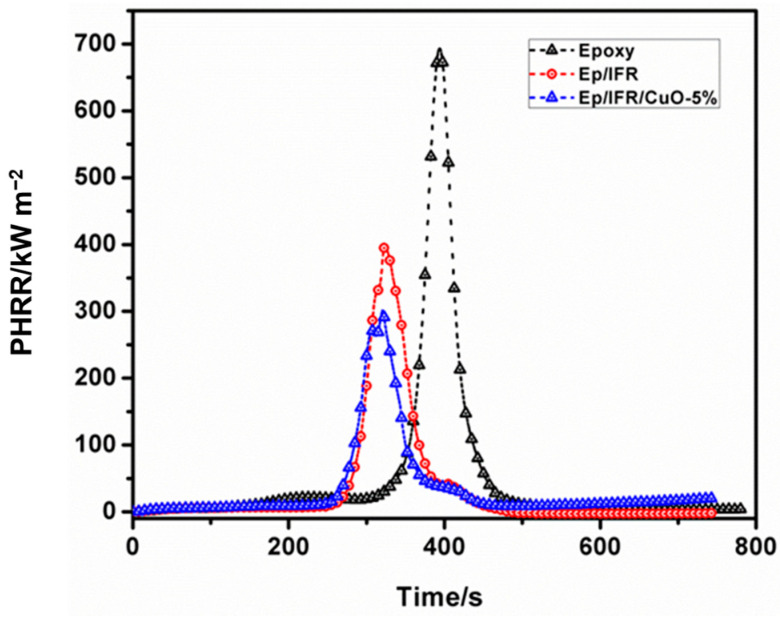
Peak heat release rate curves of epoxy, Ep/IFR, and Ep/IFR/CuO-5% samples.

The thermal stability of pure epoxy resin, Ep/IFR, and Ep/IFR/CuO-5% are represented in Figure 3 and Table 4. The data show that pure epoxy burned completely at above 510 °C. Pure epoxy showed initial mass loss during degradation at 325.8 °C (5% weight loss), whereas Ep/IFR coatings showed higher thermal stability than pure epoxy as EP/IFR coating was remained as 22.3% of char residue. The Ep/IFR coatings showed initial thermal degradation at 282 °C and showed 22.3% of char residue at 800 °C. Above 300 °C, APP started to decompose to produce dehydrating agents such as phosphoric acid, which removed the water residue from ETPMP. APP and ETPMP can easily participate in phosphor-ester bond formation. These –OH groups of polymer gently react with phosphoric acid to produce a phosphor-carbonaceous char layer, which can strongly inhibit the oxygen flow and transfer of heat. The Ep/IFR/CuO-5% coatings showed a higher percentage of char residue at 800 °C than all samples. As Figure 4 shows, the addition of CuO remarkably increased the char residue percentage from 22.3% to 25.4% for Ep/IFR/CuO-5% coatings. In the presence of CuO, APP and ETPMP rapidly undergo an esterification reaction to provide a dense char layer. The CuO catalytic action with the IFR system is shown in Scheme 1.

### 2.6. Cone Calorimeter Analysis

The combustion behavior of the polymers and materials was evaluated by the widely used cone calorimeter. 

Figure 4 and Table 5 represent the heat release rate (HRR) data for epoxy, Ep/IFR, and Ep/IFR/CuO-5% coatings. The epoxy coating burned easily and had an PHRR value of 693.5 kW m^−2^. The epoxy coating ignited at 347 s. The Ep/IFR and Ep/IFR/CuO-5% coatings have PHRR values of 397.8 and 294.8, respectively. This means the PHRR values are greatly reduced from 100% to 57.36% and 42.50% for Ep/IFR and Ep/IFR/CuO-5% coatings, respectively. 

A TTI (Time to Ignition) value provides information on the ignition parameter. The Ep/IFR and Ep/IFR/CuO-5% have lower TTI values than pure epoxy. The results show that the ignition values for Ep/IFR and Ep/IFR/CuO-5% are decreased from 347 s to 279 s and 265 s, respectively. 

The THR (Total Heat Release) data of the epoxy, Ep/IFR, and Ep/IFR/CuO-5% coatings are shown in Table 5. These data show that epoxy coating has the highest THR value of 112 MJ m^−2^, whereas the compositions of Ep/IFR and Ep/IFR/CuO-5% coatings showed a significant decrease in THR value from 112 to 73 and 42 MJ m^−2^ kg, respectively. The THR values of Ep/IFR and Ep/IFR/CuO-5% coatings were 34.83% and 62.5% lower, respectively, than epoxy coatings. 

During the combustion process, the IFR system forms a char with foam. The loading of CuO into the coating system can speed up the intumescent reaction to form a compact char. The resulting char acts as a strict barrier for the oxygen and flow of heat, greatly reducing PHRR and THR values. Among all the coatings samples, the Ep/IFR/CuO-5% coating showed the lowest PHRR, THR, and TTI values. As CuO helps in the homogenous and compact char formation, the CuO-loaded coating samples showed good combustion behavior. The results prove that CuO can be used as a synergist.

### 2.7. Morphology of the Char Residue

**Figure 5:**
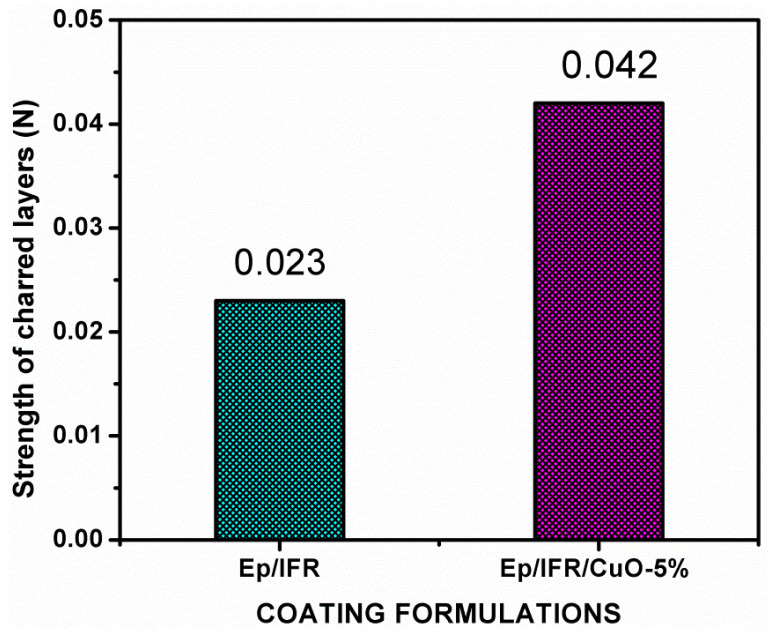
Strength analysis results of epoxy, Ep/IFR, and Ep/IFR/CuO-5% sample charred layers.

**Figure 6:**
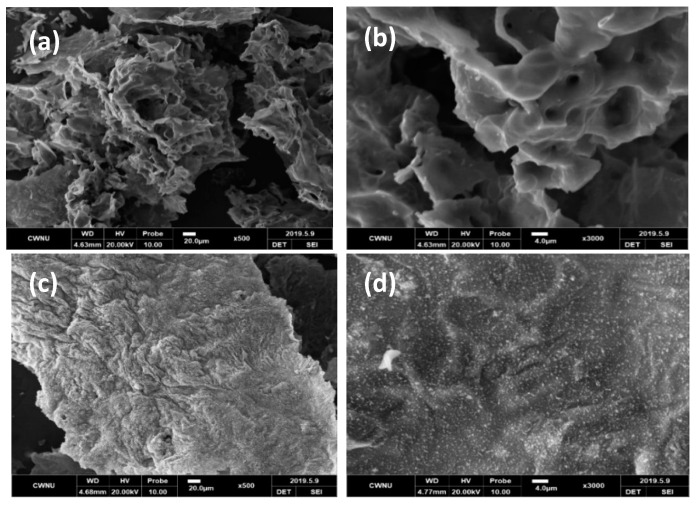
(**a**,**b**): SEM images of an Ep/IFR charred layer with different amplifications; (**c**,**d**): SEM images of Ep/IFR/CuO-5% charred layers with different amplifications.

The char residues of Ep/IFR and Ep/IFR/CuO-5% obtained in the combustion test were imaged by SEM with 500× and 3000× magnification. The SEM pictures of Ep/IFR and Ep/IFR/CuO-5% charred layers are shown in Figure 5. Figure 6a,b show that Ep/IFR char has cracks and is randomly arranged. Figure 6c,d indicate the homogeneity and smoothness of the Ep/IFR/CuO-5% char layer. The holes are formed due to the vaporization of the volatile gases. The addition of CuO can strongly interact with the IFR content and make a dense char that significantly stops the flow of heat and oxygen. We can observe that the Ep/IFR/CuO-5% char residue is very smooth without any holes and cracks. These results show that the addition of CuO can support and strengthen char formation.

### 2.8. Strength of the Residual Charred Layer

Figure 5 represents the strengths of chars of Ep/IFR and Ep/IFR/CuO-5%. The intumescent char layers from the cone calorimeter test were used to check the strengths with a compressing test machine. The strengths of Ep/IFR and Ep/IFR/CuO-5% sample chars were 0.023 N and 0.042 N. The char of Ep/IFR/CuO-5% is stronger than that of the Ep/IFR char layer because CuO can form bonds with the IFR system to make more char compact and strong. Therefore, the flow of heat transfer and oxygen can be inhibited by this strengthened char. These results strongly support CuO’s ability to be a synergist to form a tough char.

## 3. Materials and Methods

Bisphenol A (≥99%), an epoxy resin was supplied from Sigma-Aldrich (St. Louis, MO, USA). Polyamide was obtained from Sigma-Aldrich (Hamburg, Germany) and was utilized as a curing agent for epoxy resin. The commercial ammonium polyphosphate (APP, form-II, particle size 20 µm) was purchased by Samchun Company, Seoul, Korea. The solvents ethanolamine (99%), ethanol (98%), and acetone (99.5%) were bought from Sigma-Aldrich (Hamburg, Germany). The chemicals cyanuric chloride (99%), melamine (99%), piperizine (99%), copper oxide (CuO-99%, 25 nm), and sodium hydroxide (NaOH) pellets were supplied by Sigma-Aldrich (St. Louis, MO, USA). All chemicals were used as received without any further modification. 

### 3.1. Synthesis of N-Ethanolamine Triazine-Piperizine, a Melamine Polymer (ETPMP)

**Scheme 2:**
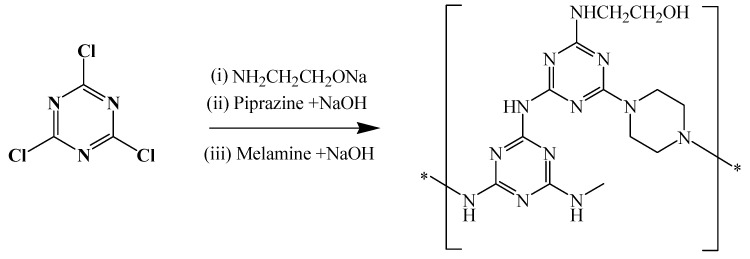
Synthesis of ETPMP. Note: * represent the repeating unit of same molecule.

The charring-foaming agent *N*-ethanolamine triazine-piperizine, a melamine polymer (ETPMP), was synthesized by a three-step nucleophilic substitution reaction as described previously [26]. The synthetic path is shown in Scheme 2. 

In step 1, 1 L of acetone and 1 mole of cyanuric chloride were transferred into a dry, clean, three-necked round-bottom flask with a 3 L capacity, and the reaction was maintained at 10 °C. One mole each of ethanolamine and NaOH were thoroughly liquefied in 1 L of distilled water in a separate flask, and the solution was transferred to the three-necked round bottom flask with continuous stirring for 2 h. At this point, the first step was complete and provided us with 2-ethanolamine-4, 6-dichloride-1, 3, 5-triazine as intermediate compounds. The remaining intermediate compounds were left in the three-necked round-bottom flask. 

Step 2 began with the complete dissolution of 0.5 moles of piperizine and 1 mole NaOH in 1 L of water in a separate flask, and this mixture of solutions was slowly transferred into the three-necked flask and left for 2 h. Next, the temperature was raised and maintained between 45 and 50 °C with continuous stirring for the next 4 h. 

In the third and last stage of the synthesis, 1 mol of NaOH and 0.3 moles of melamine were fully dissolved in 1 L of water in a separate flask. The solution was transferred into a three-necked reaction bottle. Next, the temperature of the reaction was increased again up to 70 °C for the exclusion of acetone. The step was implemented for the next 6 h to finish the reaction. The precipitate obtained was white in color and was filtered and washed thrice by ethanol to removes contamination from the final proposed product of ETPMP. Finally, 194.6 g white powder was obtained.

### 3.2. Preparation of Samples

APP and ETPMP, at a 2:1 proportion, were mixed with CuO (synergist) and added into epoxy resin (2:1) to prepare the IFR coating. CuO with APP, ETPMP were mixed in epoxy resin and dispensed into a dispersion blender for uniform mixing. Next, the prepared coatings material was brushed on selected plywood fragments for further examination and characterization. The pieces of plywood were left for two days in a ventilated room to dry completely. The composition details of the coating are listed in Table 6.

### 3.3. Fourier Transform Infrared Spectroscopy

A Nicolet Avator 360 FT-IR spectrometer instrument (Nicolet, Woodland, CA, USA) was employed to enroll the spectra of the samples at 2 cm^−1^ condition. We monitored the samples in a 4000–400 cm^−1^ range for structural information. 

### 3.4. Elemental Analysis

A Carlo Erba 1110 instrument (Strada Revoltana, Milan, Italy) was employed to check ETPMP polymer elemental composition. The obtained data are listed in Table 1. 

### 3.5. Flame Retardancy Test

The LOI tests for the samples were conducted on HC-2C Oxygen Index instrument (Jiangning Analysis, Jiangning, China). For this test, we applied 10 g of each coating compositions on plywood sheets with 130 × 6.5 × 3 mm dimensions.

A CZF-2 instrument (Jiangning Analysis, Jiangning, China) was used to record the UL-94V rankings of the testing samples. The prepared coatings were applied on 130 × 13 × 3.2 mm sized plywood pieces and dried. The results obtained are presented in Table 3.

### 3.6. Thermogravimetric Analysis (TGA) Test

A SDT Q600 V20.9 Building 20 instrument (New Castle, DE, USA) was used to check the thermal stability of the samples. The conditions of this test were 30 to 800 °C with 10 °C/min and 20 mL/min flow rate of nitrogen gas. To conduct this test, we took each sample at 5–10 mg weight.

### 3.7. Combustion Test

All combustion data were taken from the cone-calorimeter instrument (Fire Testing Technology, West Sussex, UK) that was used to perform the combustion test of all samples. The samples were placed horizontally on the sample holder. The sample was exposed to 35 kW m^−2^ of external heat flux.

### 3.8. Scanning Electron Microscopy

A scanning electron microscope JSM-6360 (Japan Electron Optics Laboratory Co., Ltd., Tokyo, Japan) was employed to analyze the morphological differences of the char layers. A gold layer was deposited on the char surface for the sputtering process. An acceleration voltage of 15 kV was maintained during the experiment. 

### 3.9. Strength of the Residual Charred Layer

We performed this test using an HY-0230 machine (Jiangning Analysis, Jiangning, China). For this test, we kept char on a circular platform, slightly increased the pressure on the char layer, and recorded where the char layer was damaged.

## 4. Conclusions

A new triazine derivative polymer *N*-ethanolamine triazine-piperizine, a melamine polymer (ETPMP), was prepared successfully. FTIR and elemental analysis data support its structural confirmation. Various IFR-Epoxy-CuO coating formulations were prepared. The loading of a small quantity of CuO into the Ep/IFR coating system can effectively increase the LOI value and reach UL-94V rankings. The LOI and UL-94V data show that the optimum amount of CuO is 5%. The TGA study showed that CuO can improve the thermal stability and char residue percentage of the samples at high temperatures. Cone calorimeter data confirm that CuO can effectively decrease the heat-releasing property. The SEM and strength analysis data prove that adding CuO at a suitable dosage can greatly encourage the formation of a uniform and compact charred layer in the burning environment of this coating. In conclusion, CuO exerts a positive influence on the epoxy headed intumescent flame retardant coatings. It is envisaged that the ecofriendly and non-toxic Copper Oxide on epoxy coatings could be exploited to develop a fire-retardant coating for the protection of inflammable materials.

## Figures and Tables

**Table 1:** Elemental analysis.

Sample	C	N	O	H
ETPMP (Calculated %)	41.74	48.69	4.63	4.92
ETPMP (Found %)	41.52	48.78	4.58	4.68

**Table 2:** TGA results of ETPMP.

Sample	Char Residue (%)	
	700 °C	800 °C
ETPMP	39.08%	35.58%

**Table 3:** Flame retardancy results of the samples.

Sample	LOI (%)	UL-94V
Epoxy	21.2	NR
Ep/IFR	28.7	V-0
Ep/IFR/CuO-1%	30.2	V-0
Ep/IFR/CuO-3%	32.7	V-0
Ep/IFR/CuO-5%	34.2	V-0
Ep/IFR/CuO-7%	32.6	V-0
Ep/IFR/CuO-9%	30.9	V-0

**Table 4:** Thermo-gravimetric results of the coatings.

Sample	Initial Degradation Temperature (Based On 5% Weight Loss)	Char Reside (%)
Epoxy	325.8 °C	00
Ep/IFR	282 °C	22.3
Ep/IFR/CuO-5%	280 °C	25.4

**Table 5:** Cone-calorimetric results of the coatings.

Sample	TTI (s)	PHRR/k W m^−2^	THR/MJ m^−2^
Epoxy	347	693.5	112
Ep/IFR	279	397.8	73
Ep/IFR/CuO-5%	265	294.8	42

**Table 6:** Composition of the samples.

Sample	Epoxy (%*w*/*w*)	APP (%*w*/*w*)	ETPMP (%*w*/*w*)	CuO (%*w*/*w*)
Epoxy	100	0	0	0
Ep/IFR	70	20	10	0
Ep/IFR/CuO-1%	69	20	10	1
Ep/IFR/CuO-3%	67	20	10	3
Ep/IFR/CuO-5%	65	20	10	5
Ep/IFR/CuO-7%	63	20	10	7
Ep/IFR/CuO-9%	61	20	10	9
